# Supplementary material for: Graphlet-based Characterization of Directed Networks
Source: Sci Rep. 2016 Oct 13;6:35098. doi: 10.1038/srep35098 (PMC5062067; doi:10.1038/srep35098)
Supplement: Supplementary Information [file srep35098-s1.pdf]

# Graphlet-based Characterization of Directed Networks: Supplementary Material

**Anida Sarajlić[1,†], Noël Malod-Dognin[2,†], Ömer Nebil Yaveroğlu[3], and Nataša Pržulj[2,*]**

[1]Department of Computing, Imperial College London, SW7 2AZ London, United Kingdom
[2]Department of Computer Science, University College London, WC1E 6BT London, United Kingdom
[3]Google UK, London, United Kingdom
[†]These authors contributed equally
[*]natasa@cs.ucl.ac.uk

## Generating random and real world directed networks

In the main manuscript, we present results obtained on synthetic directed networks from six random models, as well as on real world networks from biology and from economy. We detail here how we generated these networks.

### The six directed network random models

**Directed Erdös-Rènyi random model (ER)** represents uniformly distributed random interactions[1]. A directed ER network is generated by fixing the number of nodes in the network, and then by randomly adding directed edges (whose directionality are chosen randomly with probability of 0.5 for each direction) between uniformly chosen pairs of nodes until a given density is reached.

**Directed Barabàsi-Albert Scale-free models (SFBA-Sink and SFBA-Source).** Scale free model, also called preferential attachment, generates networks based on the "rich-gets-richer" principle[2]. Scale-free topology is characterized by power law degree distributions[2]. We use the directed preferential attachment model[3]. Starting from a small seed network having $m_0$ nodes, Directed SFBA networks are constructed by an iterative process, in which each iteration randomly performs one of following three possible actions. With probability $\alpha$, a new node $v$ is added to the network and is linked with an existing node $w$ (chosen based on the existing nodes' in-degrees) by a directed edge from $v$ to $w$. Similarly, with probability $\beta$, a new node $u$ is added to the network and is linked with existing node $v$ (chosen based on the existing nodes' out-degrees) by a directed edge from $v$ to $u$. Finally, there is a probability $\gamma$ of adding a directed edge from an existing node $v$ (chosen based on the existing nodes' out-degrees) to an existing node $w$ (chosen based on the existing nodes' in-degrees). The sum of probabilities $\alpha+\beta+\gamma$ equals 1. This iterative process is repeated until the desired numbers of nodes is obtained. To achieve desired network densities, we allow the node being added to the network to be connected to $m \leq m_0$ existing nodes, analogous to Albert-Barabasi model[2]. We do not allow duplicated edges. With this generator, we create two sets of SFBA networks. By using $\alpha = 0.1$, $\beta = 0.3$ and $\gamma = 0.6$, we create *SFBA-Sink* networks whose topology are characterized by sink hubs (node having large number of incoming directed edges). Similarly, by using $\alpha = 0.3$, $\beta = 0.1$ and $\gamma = 0.6$, we create *SFBA-Source* networks whose topology are characterized by source hubs (node having large number of outgoing directed edges).

**Scale-free model with gene duplication and divergence (SF-GD)** is a scale-free model that mimics the gene duplication and the gene divergence processes from biology[4]. We implement the directed model as follows. In the duplication step, a node in the network is selected at random and a new node is created together with the connections to/from nodes that the "parent" node had (e.g., if there is a directed edge from the parent node to node $x$, the new node also has a directed edge toward node $x$). A directed edge between the new node and his parent is added with probability $p$, whose directionality is decided randomly with probabilities of 0.5 for both directions. The mutation step is imitated in a way that each directed edge that the new node "inherits" from its parent node is deleted with the probability $q$. This procedure is repeated until the desired number of nodes is obtained. In our implementation, we start with probabilities $p = q = 0.5$. When the desired number of nodes is reached, we check the density of the obtained network. If the density is lower or greater than the desired value, we decrease or increase the value $q$ by value $q_{step}$ respectively, starting with the value $q_{step} = 0.1$. We then repeat the process of generating the network. In case we "skipped" the desired network density by decreasing or increasing the value of $q$ by the value of $q_{step}$ in the previous iteration, we adjust the value of $q_{step}$ to $q_{step}/2$, and repeat the process until the desired network density is obtained.

**Geometric model (GEO)** represents the proximity relationship between uniformly distributed points in a $d$-dimensional space[5]. We generate directed GEO networks by uniformly distributing the desired number of nodes (points) in a 3-dimensional unit cube. Then, two nodes are connected by a directed edge, whose directionality is randomly chosen with probability of 0.5 for each direction, if the Euclidean distance between the corresponding points is smaller than a distance threshold $r$. The distance threshold is chosen so as to obtain the desired directed edge density.

**Geometric models with gene duplication (GEO-GD)** is a geometric model that mimics the gene duplication process in biology[6]. We generate directed GEO-GD networks as follows: (1) A small number of nodes are distributed randomly in space—seed network; (2) Each new node is introduced as a duplicated node of a randomly selected node in the network; (3) The new node is moved randomly from its *parent* node in the metric space, at a random distance smaller or equal to $2 \times r$, where $r$ is the distance threshold used to generate GEO networks. Once the desired number of nodes is obtained, directed edges between nodes are created following the rules of the directed geometric network model.

## Real world directed networks

**Metabolic networks** represent bio-chemical reactions between enzymes and metabolites inside a cell. Using the organism-specific pathway data available from KEGG/PATHWAY database[7] in December 2014, we reconstructed the directed metabolic networks of all Eukaryotes (299 species). KEGG/PATHWAY database maintains the molecular interaction and reaction relations for each organism specific pathway and provides information on pathways with the reactions (links) between enzyme coding genes and metabolites. Links can be directed or undirected, depending on the chemical reversibility of a specific reaction. We consider only the directed links and construct directed metabolic networks where the nodes correspond to enzyme coding genes. A directed link between two enzymes in a metabolic network denotes that one enzyme catalyses a reaction whose product is a substrate for a reaction catalysed by the other enzyme. Note that we use terms gene and enzyme for the nodes interchangeably, as the nodes in the network correspond to enzyme coding genes. The sizes of metabolic networks vary, with number of nodes mainly being between 500 to 2000 and edge densities in the range between 0.5% and 1%.

We download taxonomic classification of Eukaryotes from KEGG, which classifies Eukaryotic species according to (from the most generic to the most specific) Kingdom, Sub-Phylum and Class. To annotate enzyme coding genes with their biological functions, we use Gene Ontology (GO)[8]. A single gene can be annotated with more than one GO term, while GO term dependencies are described as a GO hierarchy. In particular, GO terms can represent biological processes (BP), molecular functions (MF) and cellular components (CC). We downloaded the gene-to-GO term mappings from NCBI [1] in March 2015. We only consider the experimentally confirmed GO annotations, *i.e.*, those with experimental evidence codes.

**World trade networks** represent trading relations between countries. Using yearly trade data from the United Nations Commodity Trade Statistics (UN Comtrade) database [2], we generated 52 directed trade networks, one for each year between 1962 and 2013. We first generated weighted networks where directed edges are weighted by the corresponding trade amounts. Each weighted network is converted into an unweighed network by removing the lowest weighted edges until 90% of the total trade amount in the networks remains.

We obtain the economic indicators of country wealth from PENN World Table (PENN) [3] and International Monetary Fund World Economic Outlook Database (WEO) [4]. When needed, all prices are expressed in 2005 US Dollars. We focus on the following nine attributes:

- Population size, which is the reported population of a country in a given year, from PENN:POP.
- Employment size, which is the number of employed persons, from PENN:EMP.
- Gross Domestic Product (GDP), from PENN:RGDPNA.
- GDP per capita, which is the GDP of a country reported to its population size, from PENN:RGDPNA divided by PENN:POP.
- Debt, which is the debt of a country, from WEO:BCA.
- Debt per capita, which is the debt of a country reported to its population size, from WEO:BCA divided by PENN:POP.
- Import, which is the total import expenses of a country, from PENN:CSH_M multiplied by PENN:RGDPNA.

[1] ftp://ftp.ncbi.nlm.nih.gov/gene/DATA/gene2go.gz
[2] Data collected in August 2014, from http://comtrade.un.org/
[3] Version 7.1, downloaded in July 2014, from https://pwt.sas.upenn.edu/
[4] downloaded in July 2014 from http://www.imf.org/

- Export, which is the total export incomes of a country, from PENN:CSH_X multiplied by PENN:RGDPNA.
- Capital Stock, which is the total capital of a country, from PENN:RKNA.

Economic attributes from WEO are available only from 1980, and economic attributes from PENN are available only until 2011. Thus, experiments using economic attributes are computed on the subset of 32 world trade networks from 1980 to 2011.

## Directed automorphism orbits

An *isomorphism* $g$ from directed network $X$ to directed network $Y$ is a bijection of nodes of $X$ to nodes of $Y$ such that $(u,v)$ is a directed edge (from $u$ to $v$) of $X$ if and only if $(g(u),g(v))$ is a directed edge (from $g(u)$ to $g(v)$) of $Y$. An *automorphism* is an isomorphism from a directed network to itself, and it highlights the symmetries in the directed network's structure. The automorphisms of a network $X$ form a group, called the automorphism group of $X$, and is commonly denoted by $Aut(X)$. If $u$ is a node of graph $X$, then the *automorphism orbit* of $u$ is $Orb(u) = \{v \in V(X) | v = g(u) \text{ for some } g \in Aut(X)\}$, where $V(X)$ is the set of nodes of graph $X$. An automorphism orbit (orbit, for brevity) groups together nodes having identical wiring pattern.

## Measuring the topological similarity between nodes in directed networks

We extend the graphlet degree vector similarity[9] with our directed graphlets to compare the topology around the nodes in our directed networks in the following way. The similarity between the $i^{th}$ directed graphlet degrees of nodes $u$ and $v$ is computed as:

$$D_i(u,v) = w_i \times \frac{|log(DGDV_u[i]+1) - log(DGDV_v[i]+1)|}{log(max(DGDV_u[i], DGDV_v[i])+2)}, \tag{1}$$

where $w_i$ is an orbit-specific weight that reduces the influence of dependent orbits. The value of $w_i$ is based on $o_i$, which is the number of orbits that affects orbits $i$ including itself; for example, if a node touches orbit 9, it also touches orbits 0 and 1, henceforth $o_9 = 3$. This leads to the following values of $o_i$:

- 1 for $i \in$ {0,1},
- 2 for $i \in$ {2, 4, 5, 6, 7, 8, 10, 11},
- 3 for $i \in$ {3, 9, 12, 13, 16, 17, 20, 21, 24, 25, 28, 29, 30, 31, 32, 33, 36, 60, 64, 68, 72, 118, 120, 125, 128},
- 4 for $i \in$ {19, 22, 23, 27, 35, 38, 44, 45, 47, 48, 52, 56, 57, 61, 63, 66, 67, 70, 73, 74, 76, 77, 78, 80, 85, 87, 91, 92, 95, 96, 100, 105, 113, 115},
- 5 for $i \in$ {14, 15, 18, 26, 34, 37, 42, 43, 49, 50, 53, 54, 58, 59, 62, 65, 69, 71, 88, 90, 93, 94, 99, 103, 107, 111, 121, 122, 126, 127},
- 6 for $i \in$ {39, 40, 41, 46, 51, 55, 75, 79, 81, 82, 83, 84, 86, 89, 97, 98, 101, 102, 104, 106, 108, 109, 110, 112, 114, 116, 117, 119, 123, 124}.

$w_i$ is then computed as:

$$w_i = 1 - \frac{log(o_i)}{log(129)}. \tag{2}$$

The *Directed graphlet Degree Vector Similarity* (DGDVS) is calculated as:

$$DGDVS(u,v) = 1 - \frac{\sum_{i=0}^{128} D_i(u,v)}{\sum_{i=0}^{128} w_i}. \tag{3}$$

## Measuring the topological similarity between directed networks

In the main manuscript, we detail how we generalized three graphlet based network distances (RGD, GDDA and GCD) to directed networks. Here, we detail how we generalized two traditional network distances to directed networks: degree distribution distance and spectral distance.

**In- and out-degree distribution distances.** The in-degree distribution of the network $G$ is encoded in a vector $D_G$, whose $i^{th}$ entry is the number of nodes in $G$ having in-degree equal to $i$. The in-degree distribution distance between two networks $G_1$ and $G_2$ is defined as the Euclidean distance between their in-degree distribution vectors:

$$d_d(G_1, G_2) = \sqrt{\sum_i (D_{G_1}[i] - D_{G_2}[i])^2}. \quad (4)$$

We alternatively considered using out-degree instead of in-degrees, but the corresponding out-degree distribution distance achieved almost identical classification results as the ones of the in-degree distribution distance.

**Directed Spectral distance.** Spectral network theory explains the topology a network using the eigenvalues and eigenvectors of matrices associated to the network, such as its adjacency matrix or Laplacian matrix[10]. The eigen-decomposition of the Laplacian matrix of $G$ is $L = \phi \lambda_G \phi^T$ where $\lambda_G = diag(\lambda_G^1, \lambda_G^2, ..., \lambda_G^n)$ is the diagonal matrix with the ordered eigenvalues as elements and $\phi = (\phi_1|\phi_2|...|\phi_n)$ is the matrix with the ordered eigenvectors as columns. The spectrum of network $G$ is the set of its eigenvalues $S_G = \{\lambda_G^1, \lambda_G^2, ..., \lambda_G^n\}$, where $\lambda_G^1 \leq \lambda_G^2 \leq ... \leq \lambda_G^n$.

Wilson and Zhu[10] compare various spectral distance measures that are defined from different types of matrices, showing that the spectral distance between the Laplacian matrices of two networks is the best measure for classification and clustering purposes. Later on, Thorne and Stumpf[11] also use the spectral distance of Laplacian matrices for the analysis of the evolution in protein interaction networks. In parallel to these studies, we chose spectral distance from Laplacian matrices as the benchmark representing the performance of spectral distance measures against graphlet based distances. The spectral distance between two networks $G$ and $H$ is defined as the Euclidean distance between their spectra[10]:

$$Spectral(G, H) = \sqrt{\sum_i (\lambda_G^i - \lambda_H^i)^2}. \quad (5)$$

When the spectra of two networks are of different sizes, 0 valued eigenvalues are added into the smaller spectrum while preserving the correct magnitude ordering.

However, in the directed case, adjacency and Laplacian matrices are not symmetric, so their spectral decomposition may have complex eigenvalues. To compute spectral distance on directed networks, we represent a directed network with its symmetric normalized Laplacian matrix $L_n$. It is based on the reciprocal square of the incidence matrix of a network, $S$:

$$S[u][e] = \begin{cases} 1/\sqrt{d(u)} & \text{if the directed edge } e = (u,v) \text{ is in the network,} \\ -1/\sqrt{d(u)} & \text{if the directed edge } e = (v,u) \text{ is in the network,} \\ 0 & \text{otherwise.} \end{cases} \quad (6)$$

Then, the symmetric normalized laplacian matrix, $L_n$, is defined as:

$$L_n = S \times S^T, \quad (7)$$

where $S^T$ is the transpose of $S$.

## Dependencies between graphlet degrees

The statistics of different orbits on directed graphlets are not independent of each other. The reason behind this is the fact that smaller graphlets are induced sub-graphs of larger graphlets. We report 23 such non-redundant dependencies in Supplementary Table 1. Note that these redundancy formulas are only valid for networks without anti-parallel directed edges, and thus, can not be used to remove redundant orbits. For example, let the number of orbits 0 that touches a node $n$ in a network without anti-parallel directed edges be $C_0$. There are $\binom{C_0}{2}$ pairs of orbits 0 that touch node $n$. Each pair can result either in the count of one orbit 6, if the corresponding end-nodes are not connected, or in the count of one orbit 11 if the two end-nodes are connected by a directed edge. Thus, $\binom{C_0}{2} = C_6 + C_{11}$, as presented in Table 1. However, if anti-parallel directed edges are allowed, this results in a third cases accounting for the count of two orbits 11, which invalidates the previously presented redundancy formula.

### *Brokerage and peripheral scores*

We use the first canonical variate, which relate the economic wealth of a country (GDP, capital stock and debt) with brokerage positions in the WTN, to derive scores measuring the brokerage power or the peripherality of a country from its node roles in the trade network of a given year. These scores use the loadings of broker and peripheral roles, which are the Pearson correlation betweens the node roles (graphlet degrees) and the linear combination of node roles from the canonical variate. For a country $u$ and its directed graphlet degree vector $DGDV_u$ in the WTN for a given year, these scores are defined as follows.

$$\begin{aligned} Brokerage(u) = \; & 0.903 \times DGDV_u[51] + 0.907 \times DGDV_u[55] + 0.907 \times DGDV_u[59] \\ & +0.901 \times DGDV_u[63] + 0.897 \times DGDV_u[67] + 0.916 \times DGDV_u[71] \\ & +0.904 \times DGDV_u[75] + 0.912 \times DGDV_u[79] \end{aligned} \quad (8)$$

| | | | |
|---|---|---|---|
| 1. | $\binom{C_0}{2}$ | = | $C_6 + C_{11}$ |
| 2. | $\binom{C_0}{1} \cdot \binom{C_1}{1}$ | = | $C_3 + C_9 + C_{12}$ |
| 3. | $\binom{C_1}{2}$ | = | $C_8 + C_{10}$ |
| 4. | $\binom{C_2}{1} \cdot \binom{C_0 - 1}{1}$ | = | $C_{27} + C_{57} + C_{73} + 2 \cdot C_{113} + C_{107} + C_{111} + 2 \cdot C_{48} + C_{42}$ |
| 5. | $\binom{C_1}{1} \cdot \binom{C_2}{1}$ | = | $C_{58} + C_{50} + C_{40} + C_{39} + C_{82} + C_{98} + C_{102} + C_{14} + C_{83}$ |
| 6. | $\binom{C_0}{1} \cdot \binom{C_5}{1}$ | = | $C_{26} + C_{65} + C_{49} + C_{40} + C_{46} + C_{102} + C_{86} + C_{89} + C_{98}$ |
| 7. | $\binom{C_5}{1} \cdot \binom{C_1 - 1}{1}$ | = | $C_{22} + C_{74} + C_{66} + 2 \cdot C_{44} + C_{43} + 2 \cdot C_{95} + C_{99} + C_{103}$ |
| 8. | $\binom{C_7}{1} \cdot \binom{C_0 - 1}{1}$ | = | $C_{23} + C_{61} + C_{77} + C_{42} + 2 \cdot C_{45} + C_{107} + 2 \cdot C_{92} + C_{111}$ |
| 9. | $\binom{C_7}{1} \cdot \binom{C_1}{1}$ | = | $C_{18} + C_{62} + C_{54} + C_{46} + C_{41} + C_{86} + C_{110} + C_{106} + C_{89}$ |
| 10. | $\binom{C_4}{1} \cdot \binom{C_0}{1}$ | = | $C_{15} + C_{53} + C_{69} + C_{39} + C_{41} + C_{83} + C_{106} + C_{110} + C_{82}$ |
| 11. | $\binom{C_4}{1} \cdot \binom{C_1 - 1}{1}$ | = | $C_{19} + C_{78} + C_{70} + C_{43} + 2 \cdot C_{47} + C_{99} + 2 \cdot C_{115} + C_{103}$ |
| 12. | $\binom{C_3}{1} \cdot \binom{C_1 - 1}{1}$ | = | $2 \cdot C_{37} + 2 \cdot C_{59} + C_{79} + C_{55} + C_{97} + C_{112} + C_{116} + C_{81}$ |
| 13. | $\binom{C_3}{1} \cdot \binom{C_0 - 1}{1}$ | = | $2 \cdot C_{34} + C_{51} + C_{75} + 2 \cdot C_{71} + C_{101} + C_{108} + C_{84} + C_{114}$ |
| 14. | $\binom{C_6}{1} \cdot \binom{C_0 - 2}{1}$ | = | $3 \cdot C_{32} + 2 \cdot C_{67} + C_{96} + C_{87} + C_{100}$ |
| 15. | $\binom{C_6}{1} \cdot \binom{C_1}{1}$ | = | $C_{34} + C_{75} + C_{51} + C_{94} + C_{90} + C_{104}$ |
| 16. | $\binom{C_8}{1} \cdot \binom{C_0}{1}$ | = | $C_{37} + C_{55} + C_{79} + C_{88} + C_{93} + C_{109}$ |
| 17. | $\binom{C_8}{1} \cdot \binom{C_1 - 2}{1}$ | = | $3 \cdot C_{30} + 2 \cdot C_{63} + C_{85} + C_{91} + C_{105}$ |
| 18. | $\binom{C_9}{1} \cdot \binom{C_0 - 1}{1}$ | = | $C_{51} + C_{104} + 2 \cdot C_{90} + C_{84} + C_{101} + 2 \cdot C_{121} + C_{117} + C_{124}$ |
| 19. | $\binom{C_9}{1} \cdot \binom{C_1 - 1}{1}$ | = | $C_{55} + C_{81} + C_{112} + C_{109} + 2 \cdot C_{88} + C_{119} + 2 \cdot C_{122} + C_{123}$ |
| 20. | $\binom{C_{10}}{1} \cdot \binom{C_1 - 2}{1}$ | = | $C_{63} + 2 \cdot C_{105} + 2 \cdot C_{85} + 2 \cdot C_{91} + 3 \cdot C_{125} + 3 \cdot C_{118}$ |
| 21. | $\binom{C_{10}}{1} \cdot \binom{C_0}{1}$ | = | $C_{59} + C_{97} + C_{81} + C_{116} + C_{112} + C_{127} + C_{122} + C_{119} + C_{123}$ |
| 22. | $\binom{C_{11}}{1} \cdot \binom{C_0 - 2}{1}$ | = | $C_{67} + 2 \cdot C_{87} + 2 \cdot C_{100} + 2 \cdot C_{96} + 3 \cdot C_{128} + 3 \cdot C_{120}$ |
| 23. | $\binom{C_{11}}{1} \cdot \binom{C_1}{1}$ | = | $C_{71} + C_{84} + C_{108} + C_{114} + C_{101} + C_{121} + C_{126} + C_{124} + C_{117}$ |

**Table 1. The 23 non-redundant dependencies between directed graphlet degrees.** These equations come from the observation that combining two orbits from the left-hand-sides can only results in orbits from the right-hand-sides.

$$Peripherality(u) = 0.286 \times DGDV_u[52] + 0.279 \times DGDV_u[56] + 0.284 \times DGDV_u[60] + 0.275 \times DGDV_u[64] + 0.279 \times DGDV_u[68] + 0.272 \times DGDV_u[72] + 0.281 \times DGDV_u[76] + 0.274 \times DGDV_u[80] \quad (9)$$

Supplementary Figure 1 presents the brokerage and peripheral scores of the strongest brokers (United States of America, United Kingdom, Germany, France and China, panels a and b) and of emerging economies (Brazil, Russia, India, Turkey and Indonesia, panels c and d). To further study how China integrated the world trade network, we use import-specific and export-specific (according to the directionality of the trade between the broker role and the peripheral role) variants of the brokerage score:

$$Import\_Brokerage(u) = 0.907 \times DGDV_u[55] + 0.901 \times DGDV_u[63] + 0.916 \times DGDV_u[71] + 0.912 \times DGDV_u[79] \quad (10)$$

$$Export\_Brokerage(u) = 0.903 \times DGDV_u[51] + 0.907 \times DGDV_u[59] + 0.897 \times DGDV_u[67] + 0.904 \times DGDV_u[75] \quad (11)$$

Supplementary Figure 1 presents the import- and export-specific brokerage scores of emerging economies (Brazil, Russia, India, Turkey and Indonesia, panels e and f).

## Supplementary Figures

## References


1. Erdös, P. & Rényi, A. On random graphs. *Publicationes Mathematicae* **6**, 290–297 (1959).
2. Barabási, A.-L. & Albert, R. Emergence of scaling in random networks. *Science* **286**, 509–512 (1999).
3. Bollobás, B., Borgs, C., Chayes, J. & Riordan, O. Directed scale-free graphs. In *Proceedings of the Fourteenth Annual ACM-SIAM Symposium on Discrete Algorithms*, 132–139 (Society for Industrial and Applied Mathematics, 2003).
4. Vázquez, A., Flammini, A., Maritan, A. & Vespignani, A. Modeling of protein interaction networks. *Complexus* **1**, 38–44 (2002).
5. Penrose, M. Random geometric graphs. *Oxford Studies in Probability* **5** (2003).
6. Pržulj, N., Kuchaiev, O., Stevanovic, A. & Hayes, W. Geometric evolutionary dynamics of protein interaction networks. In *Pacific Symposium on Biocomputing*, vol. 2009, 178–189 (World Scientific, 2010).
7. Kanehisa, M. Toward pathway engineering: a new database of genetic and molecular pathways. *Science & Technology Japan* **59**, 34–38 (1996).
8. Ashburner, M., Ball, C. A., Blake, J. A. *et al.* Gene ontology: tool for the unification of biology. *Nature Genetics* **25**, 25–29 (2000).
9. Milenković, T. & Pržulj, N. Uncovering biological network function via graphlet degree signatures. *Cancer Informatics* **6**, 257 (2008).
10. Wilson, R. C. & Zhu, P. A study of graph spectra for comparing graphs and trees. *Pattern Recognition* **41**, 2833–2841 (2008).
11. Thorne, T. & Stumpf, M. P. Graph spectral analysis of protein interaction network evolution. *Journal of The Royal Society Interface* **9**, 2653–2666 (2012).

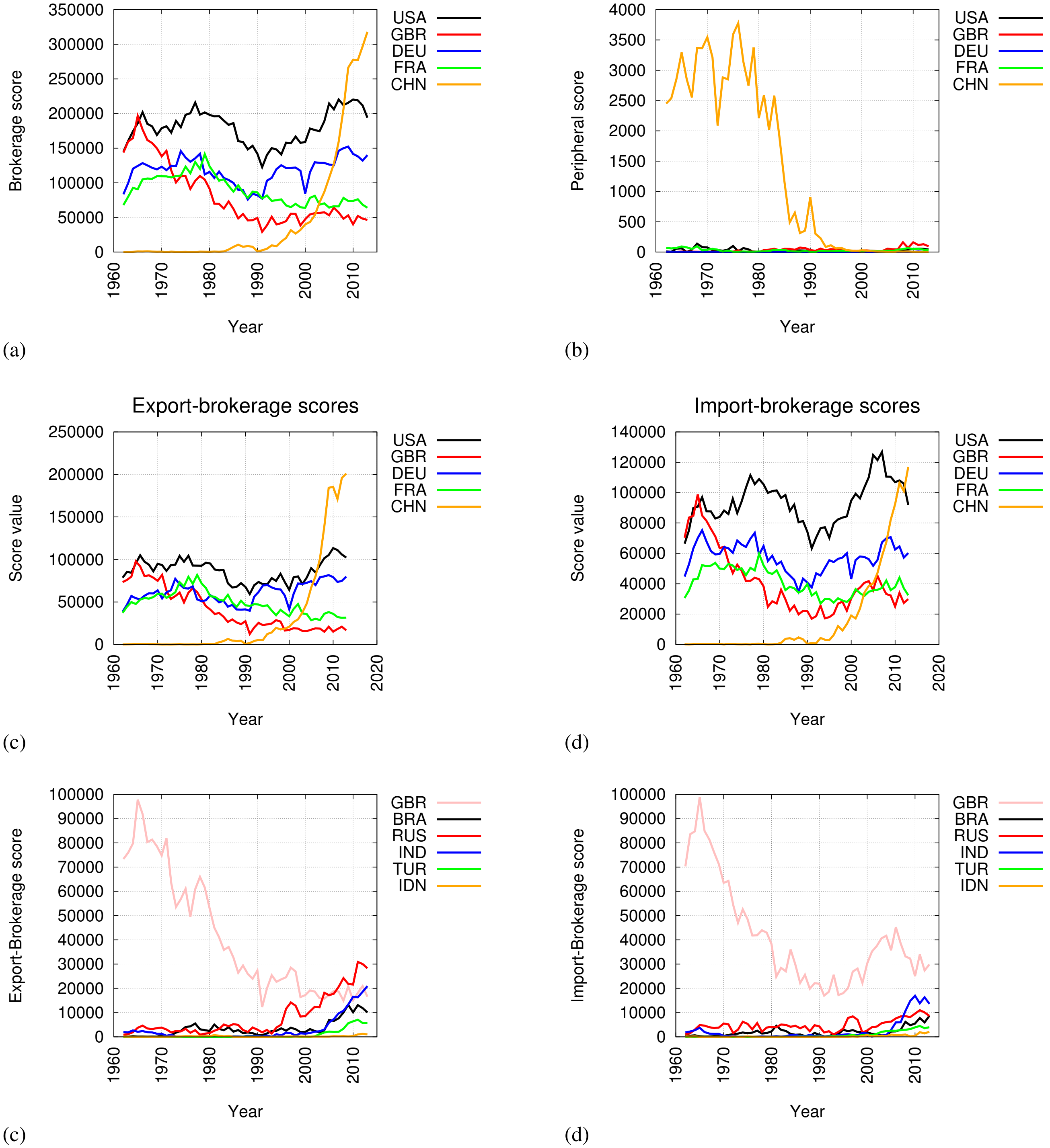


**Supplementary Figure 1: Tracking the dynamics of WTN.** (a) and (b) Brokerage and peripheral scores of the United States of America (USA), United Kingdom (GBR), Germany (DEU), France (FRA) and China (CHN). (c) and (d) Export-specific and import-specific brokerage scores of the United States of America (USA), United Kingdom (GBR), Germany (DEU), France (FRA) and China (CHN). (e) and (f) Export-specific and import-specific brokerage scores of Brazil (BRA), Russia (RUS), India (IND), Turkey (TUR) and Indonesia (IDN); United Kingdom (GBR, in pink) is added for comparison purposes.

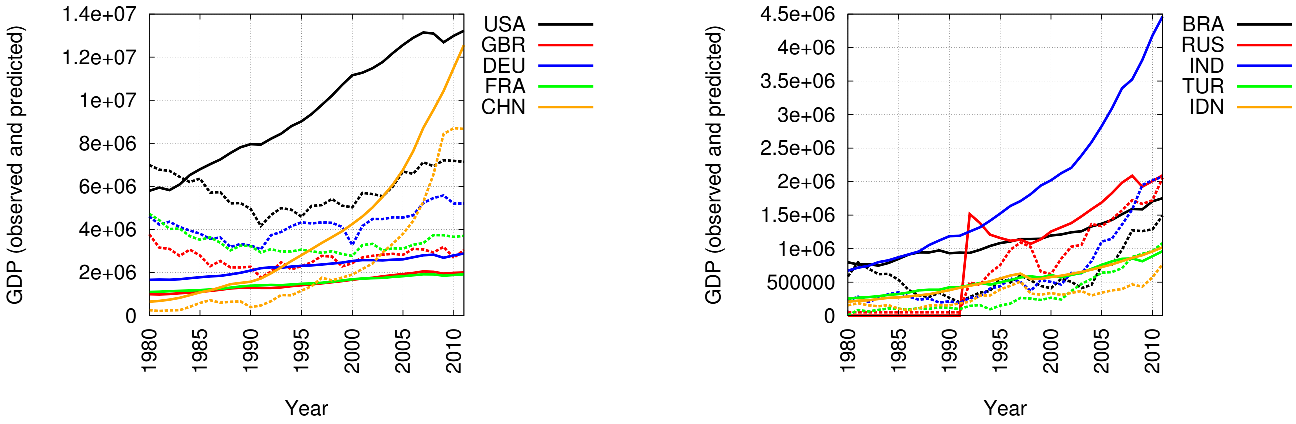


(a) (b)

**Supplementary Figure 2: Observed and predicted GDPs.** (a) For all of United States of America (USA), United Kingdom (GBR), Germany (DEU), France (FRA) and China (CHN), the observed GDPs are plotted in bold lines and the predicted GDPs (from the trade patterns of the countries) are plotted in dashed lines. (b) The same for Brazil (BRA), Russia (RUS), India (IND), Turkey (TUR) and Indonesia (IDN).

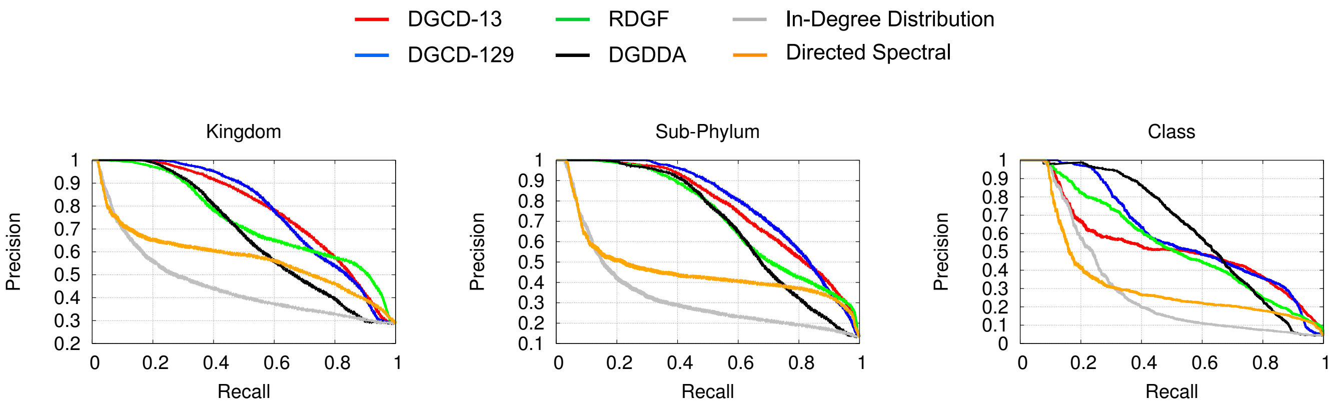


(a) (b) (c)

| | AUPR | | | AUC | | |
|---|---|---|---|---|---|---|
| Distance | Kingdom | Sub-Phylum | Class | Kingdom | Sub-Phylum | Class |
| DGCD-129 | 0.782 | **0.786** | 0.600 | 0.839 | 0.923 | 0.927 |
| DGCD-13 | **0.784** | **0.786** | 0.530 | 0.850 | **0.928** | **0.945** |
| DGDDA | 0.677 | 0.687 | **0.629** | 0.751 | 0.852 | 0.873 |
| RDGF | 0.738 | 0.718 | 0.544 | **0.854** | 0.914 | 0.934 |
| In-Degree Distribution | 0.458 | 0.335 | 0.305 | 0.635 | 0.708 | 0.755 |
| Directed Spectral | 0.574 | 0.459 | 0.339 | 0.777 | 0.863 | 0.893 |

(d)

**Supplementary Figure 3: Classifying metabolic networks.** Precision Recall curves when classifying metabolic networks according to the Kingdom (panel a), Sub-phylum (panel b) and Class (panel c) levels of KEGG's taxonomic classification of species. (d) The corresponding area under the precision-recall curves (AUPRs) and area under the ROC curves (AUCs).
